# A Comprehensive Yogic Breathing Program: Evidence for Use in Medicine

**DOI:** 10.7759/cureus.90823

**Published:** 2025-08-23

**Authors:** Jyotsna P Viveka

**Affiliations:** 1 Endocrinology and Diabetes, All India Institute of Medical Sciences (AIIMS) New Delhi, New Delhi, IND

**Keywords:** breath-mind-body technique, comprehensive yogic breathing program, mind-body medicine, sudarshan kriya yoga, yoga, yogic breathing

## Abstract

Mind-body interplay in the maintenance of health and the development of disease is important. With the escalating pace of life, increasing demands in ever-changing chaotic surroundings, and practices that help us revive within this mind-body complex are even more relevant today for preserving good health. One such commonly available practice is the Sudarshan Kriya Yoga (SKY), which is now widely available in over 180 countries. During COVID and in the post-COVID era, it has been administered online, making it more accessible. There is mounting evidence of the benefits of SKY in various body systems, like central nervous system, cardiovascular system, and respiratory system. Studies show the effect of SKY on immunity, oxidative stress, and genomic expression. It has shown benefit in autonomic functions, anxiety, depression, diabetes, addiction, substance abuse, sleep disorders, quality of life, social connectedness, and adolescent health. This review aims to compile evidence for the use of SKY in medicine and also explore the mechanism through which it acts based on published literature.

## Introduction and background

Mind-body interplay in the maintenance of health and the development of disease is important. With the escalating pace of life, increasing demands in ever-changing chaotic surroundings, information bombardment through social media requiring us to make right choices, and practices that help us establish and rest within this mind-body complex are now even more relevant for preserving good health. One such widely available practice is the Sudarshan Kriya Yoga (SKY). SKY is a sequence of breathing techniques (Ujjayi breath, Bhastrika, and Sudarshan Kriya), which constitute cyclical rhythmic breathing that harmonizes body, mind, and emotions. It can be easily and effectively practiced after proper training by a certified teacher. Once it is learned, it takes around 30 minutes of daily practice and weekly one-hour group practice. This is taught in a yogic breathing-based life skills workshop. Since the first published article on SKY in 1998 in a PubMed-indexed journal [1], evidence has been mounting on its utility in various health-related conditions. Therefore, a narrative review was planned to determine its benefits and safety.

During COVID and in the post-COVID era, it has been administered online, making it more accessible. There is evidence of the benefits of SKY in various body systems, like the central nervous system [2], cardiovascular system, and respiratory system. It has also shown benefit in autonomic functions, anxiety, depression, diabetes, addiction, substance abuse, sleep disorders, quality of life, social connectedness, adolescent health, and challenging professions like military veterans and health care professionals. Studies also show positive effect of SKY on immunity, oxidative stress, and genomic expression.

The aim of this narrative review is to compile studies on SKY from across the globe and evaluate its effectiveness. The mechanisms through which SKY has such varied benefits have also been explored based on the available literature.

## Review

Materials and methods

For this narrative review, PubMed was searched for the keyword “Sudarshan kriya yoga.” All published articles from inception till May 2025 (observational study, randomized control trials [RCT], and reviews) were taken. These were enlisted under various systems. Evidence of medical benefits and possible mechanisms of benefits of SKY, based on recently published literature, was discussed.

Central Nervous System

In an observational study, high-frequency cerebral activation was seen on electroencephalogram (EEG) following SKY. Another observation was interhemispheric synchronization. Electrical activity shifted from lower to higher frequency range with a significant increase in the gamma and beta powers following SKY [2].

Quantitative EEG interpretation revealed that there were changes in the amplitude of brain waves related to a state of relaxation and significantly improved concentration following SKY, thereby leading to restful alertness [3]. An RCT using a non-linear EEG signal processing algorithm showed improved information processing in the brain, improved performance, and reduced error in determination test scores in those practicing SKY [4].

In another RCT, there was an increase in alpha and beta energies and root mean square of the EEG signal in the SKY group, while these decreased in the control group. Also, the subjective score of work significantly reduced, and the task performance increased significantly in the group practicing SKY compared to the group not practicing [5]. The authors concluded that stress regulation by SKY has a positive effect on workload tolerance and multitasking.

Another study by the same group showed that the group practicing SKY had a change in brain rhythms that improved working memory capacity compared to the control group [6]. Stress determination test and EEG revealed a decrease in mental stress and better cognitive performance in SKY compared to the control group [7]. EEG signal-based classification before and after yoga and SKY was used by applying an artificial neuronal network to statistical parameters to classify patients as meditators and non-meditators with 87.2% accuracy [8].

In another randomized control study, SKY has also shown to enhance pre-attentive processing [9]. In a recent study from Poland, it was found that in post-COVID patients just after three interventions, SKY significantly affects brain activity by changing the amplitude of emitted brain waves, thereby improving parameters that translate into improvement of cognitive abilities such as concentration and memory [10].

Cardiovascular System

In multiple studies, practice of SKY has shown a positive effect on cardiac autonomic functions and heart rate variability (HRV) in healthy persons [11-15] and persons with diabetes [16,17], anxiety/depression [18], and post-traumatic stress disorder [19]. In an RCT of 60 healthy adults, it was observed that in the group practicing SKY, there was positive stress coping behavior, and cardiac autonomic tone showed appropriate balance [11]. This positive change was not seen in the control group and the group that received another yoga training.

In another study of healthy adults, electrocardiogram (ECG) and skin conductance level were recorded before and after long SKY. Parasympathetic activity increased, sympathetic activity decreased, and sympathovagal balance improved following long SKY. There was a decrease in skin conductance, which reflects a decrease in sympathetic activity. The authors concluded that long SKY has a beneficial effect on cardiac autonomic tone, HRV, and psychophysiological relaxation [12]. A pilot study and RCT from the United States reported improvement in vagal tone [13] and cardiac metrics of stress, respectively, following the SKY workshop [14].

In an observational study, SKY was found to enhance cardiovascular and respiratory synchronization. Highly significant reciprocal correlation was found between HRV parameters and respiration rate during long SKY [15]. The authors concluded that the practice of long SKY has the potential to influence cardiorespiratory parameters for improving the performance of both systems. Long SKY enhances oscillations in HRV that reset the autonomic system, indicative of better cardiac health, and prepares the body for better metabolic response. The authors concluded that such changes are capable of inducing resilience along with physiological, psychological relaxation, and emotional well-being.

In an RCT of 120 subjects with diabetes, change in glycemic control, cardiac autonomic functions, and quality of life with practice of SKY was studied [16,17]. Patients with type 2 diabetes were randomized into either a group that received standard treatment or the other group that received SKY training and practice. At six months post-intervention, there was significant improvement in physical, psychological, and social domains and total quality of life in the SKY group as compared with the group following standard treatment alone. There was a significant improvement in sympathetic cardiac autonomic functions in the group practicing SKY but not in the standard therapy-only group. One of the causes why sudden cardiac death is more common in diabetes compared to persons without diabetes is the occurrence of cardiac autonomic neuropathy. Since SKY practice has shown to delay progression of cardiac autonomic neuropathy, it may have important implications in the prevention of sudden cardiac deaths [16,17]. However, long-term RCTs are needed to confirm this.

In a study from Italy, improvement in cardiac autonomic control and cardiorespiratory coupling was seen in patients with anxiety and depression practicing SKY [18]. The study suggested that SKY training may be a useful non-pharmacological intervention to decrease cardiovascular risk. In an RCT done on US veterans undergoing treatment for post-traumatic stress disorder, improvement in HRV was observed in the group practicing SKY compared to those practicing cognitive processing therapy [19].

Oxidative Stress Markers

Activity of glutathione peroxidase, glutathione reductase, superoxide dismutase (SOD), catalase (oxidative enzymes), and glutathione levels have been shown to improve in persons practicing SKY [20].

In an open-label intervention study of mild hypertensive and healthy adults, there was a decrease in plasma malondialdehyde adducts, serum urea, and diastolic blood pressure on practice of SKY for two months [21]. It was observed that values within the normal range did not change, while values above the normal range came down toward normality.

Many chronic diseases have oxidative stress as a contributory factor in their pathogenesis. In another study, it was found that those practicing SKY had significantly lower levels of blood lactate and higher levels of SOD, glutathione, and catalase compared to those not practicing SKY [22]. Thus, there is evidence that regular practice of SKY leads to a better antioxidant state in the body [23].

Depression, Anxiety, and Post-Traumatic Stress Disorder (PTSD)

There are numerous reports of improvement in depression and anxiety with the practice of SKY from across the world [13-14,24-37]. In RCTs done on 74 young adults in the United States, this yogic breathing-based life skills workshop reported improvement in self-reported depression, perceived stress, emotion regulation, life satisfaction, social connectedness, conscientiousness, self-esteem, and gratitude after the workshop compared to the control group. Post workshop, the participants reported less sadness and fatigue [13,14]. SKY has shown benefit in post-traumatic stress disorders [19] and has shown to increase social connectedness [26].

In a recent feasibility study from Canada [24] for youth at risk of homelessness, depression, anxiety, and drug abuse, it was observed that it was feasible to conduct a pilot study on the utility of SKY, and there was a high retention rate. Another recent RCT from Europe reports improvements in wellness and decreased burnout among physicians who regularly practiced SKY compared to those who did not [25]. This study also brings forth that SKY is an efficacious, doable, and risk-free technique to enhance wellness and reduce burnout in physicians [25].

A study compared the efficacy of SKY in melancholia with electroconvulsive therapy (ECT) and imipramine. Patients with melancholia were randomized into three groups. They were assessed at baseline and weekly for four weeks. It was found that SKY as a first-line treatment can be a potential alternative to drugs in melancholia [38].

In a feasibility study from Canada, it was found that it is possible to conduct a clinical trial on SKY in a routine psychiatric clinic serving patients with PTSD due to a wide range of trauma [24,32]. Other studies from Canada and Sweden revealed that SKY practice decreased anxiety, stress, and depression and that a full-scale trial is possible [29,39]. During COVID and in the post-COVID period, the feasibility of online administration of SKY has also been confirmed [25,29,32].

Thus, numerous studies from Kuwait [33], Canada [24,29,32], the United States [14,19,28,30], and Europe [25] have recognized the benefits of the practice of SKY in depression, anxiety, and PTSD.

Addictions and Substance Abuse

In a recent review, it was found that there is evidence to support the integration of SKY into the management of substance use disorders, and the authors concluded that larger sample size research is imperative to establish the promising role of SKY as evidence-based medicine for the management of substance use disorders [26]. In addition to medication, psychotherapy is considered an important component of long-term opioid use disorder treatment. In a study done in rural patients with opioid use disorder in the United States, SKY was added to the standard treatment [28]. It was observed that 87.5% patients successfully completed the SKY training. The decrease in substance use cravings and depression was significant as compared to baseline. In addition, physical and social functioning as well as mental and emotional well-being significantly improved after SKY. Though the sample size of this study was small, this study opens up ground for research on the use of incorporating SKY in the management of opioid disorder.

Practice of SKY has been shown to reduce substance abuse craving [28], improve physical and emotional well-being in individuals with tobacco addiction, alcohol dependence syndrome, and substance use disorders [26]. SKY helped in the control of tobacco habit in 21% individuals who were followed up for six months of practice [40]. Authors of the above noted that it is inexpensive and easy to learn.

Sleep and Insomnia

SKY has been shown to improve insomnia [25], nocturnal sleep, and daytime wakefulness [41]. In a prospective, randomized controlled study, SKY was compared with another sitting activity/Suryanamaskar, each performed for eight weeks. Epworth sleepiness scale (ESS) is a measure of excessive daytime sleepiness. The SKY group showed significant improvement in the ESS score at four and eight weeks. No improvement was seen in the controls [41]. The authors postulated that regulated vagal nerve (parasympathetic) activity after SKY could be countering increased HRV alterations and sympathetic hyperstimulation in chronic insomnia as well as cholinergic/GABAergic dysregulation in anxiety disorders. This study adds to the evidence that SKY improves nocturnal sleep and reduces excessive daytime sleepiness (total and situational).

In a cross-sectional study of 387 adults from Singapore, it was found that the frequency of practice of SKY was directly proportional to good sleep quality [42].

Genetic Expression

As early as 2008, it was reported that SKY may modulate gene transcription [43]. That study compared gene expression in 42 SKY practitioners with 42 normal healthy adult controls, and upregulation of prosurvival and antiapoptotic genes was found in SKY practitioners as compared to normal healthy adult controls [43]. The sample size was small, and there could be a bias of self-selection.

Subsequently, other studies have also shown epigenetic effects of SKY. Practices related to SKY have been reported to reduce mRNA expression of pro-inflammatory genes and increase expression of antioxidative genes [44]. It has also been seen that telomere length increased significantly compared to baseline in those practicing SKY [45].

In another study from Norway, a change in gene expression was seen in healthy individuals practicing SKY compared to the control group [46]. This change was studied in peripheral blood mononuclear cells, was global, rapid, and significantly greater in the SKY group compared to the control group. Thus, it is evident from multiple studies that SKY and related practices positively influence gene expression [43-46].

Quality of Life (QoL)

SKY has been shown to improve QoL in multiple studies in healthy persons [11-15,37], patients with diabetes [33,47], persons living with HIV [48], individuals with post-traumatic stress disorder [29], post-menopausal women [49], patients with depression or anxiety [34], and those with opioid [50] and alcohol dependence [51]. In an RCT done in patients with type 2 diabetes, significant improvement in QoL was observed in the SKY group as compared with the group following standard treatment alone [47]. The improvement in QoL spanned over psychological, physical, and social domains as well as total QoL.

Immune Functions

SKY has a beneficial effect on immunity [23]. SKY has been shown to increase natural killer cell count significantly at 12 and 24 weeks of practice compared to baseline and at 24 weeks compared to controls [40]. Genes encoding SOD and glutathione peroxidase (which are known to have anti-inflammatory properties) are expressed more by SKY-related practices, while genes encoding IL-1β, IL-6, and TNF (which have pro-inflammatory properties) are expressed less [44].

Diabetes Mellitus

Multiple studies have shown that the practice of SKY has several benefits in patients with type 2 diabetes. Patients practicing SKY had improvement in glycemic control [17,52], QoL [33,47], cardiac autonomic functions [16,17], anxiety, depression, and insomnia [33] compared to those not practicing SKY. In another study, it was shown that patients practicing SKY were more successful in improving dietary practices and medication adherence, and there was an increasing proportion of diabetes and hypertensive patients under control in the group practicing SKY [53]. This was a small sample size study, yet this preliminary finding of improving dietary practices and adherence to medication needs to be confirmed in larger studies, as it has important implications in the control of chronic diseases like diabetes.

Health Care Personnel and Military Veterans

Due to exposure to disease, disability, death on a regular basis, and high stress levels, medical personnel are prone to burnout. This has an adverse effect on health care system. A multicentric randomized clinical trial assessed the efficacy of SKY compared to a stress management education program among physicians [25].

This online study included 129 physicians from Turkey, Germany, and Dubai who consented to practice some relaxation technique daily for two months. Compared with the control group, participants in the SKY group had significantly decreased interpersonal disengagement, stress, depression, insomnia, work exhaustion, and burnout from baseline. The SKY group also showed significant improvement in professional fulfillment. Therefore, authors concluded that SKY is an efficacious, feasible, and risk-free intervention to increase wellness, resilience, and reduce burnout among physicians [25].

Another recent prospective study was conducted among medical doctors (interns, residents, and consultants) in a tertiary care center in Nepal. Stress and self-esteem levels were assessed at baseline and follow-up of SKY practice. The scores for stress decreased, and self-esteem levels increased significantly after SKY practice, signifying a beneficial impact [54].

During the COVID-19 pandemic, to examine the efficacy of SKY for alleviating work exhaustion and influencing positivity, a study was designed among physicians. It was found that immediately after SKY, physicians experienced a significant improvement in professional fulfilment and reduced work exhaustion. Authors concluded that SKY can aid medical personnel in maintaining well-being when faced with unexpected challenges [55,56].

Another stressful profession in which there are studies on SKY is that of military veterans. Multiple studies have shown the benefits of SKY among military veterans in the United States [19,30,31] and Canada [29]. Emotional regulation and HRV improved [19], and it was also observed that SKY can be virtually delivered, making it feasible [29].

In a parallel randomized controlled non-inferiority trial, it was found that SKY is as effective as cognitive processing therapy for managing PTSD among US military veterans and merits further consideration as a treatment for PTSD [30,31].

Wellness and Optimism

In an RCT conducted in Sweden, healthy volunteers were randomized into either a group that practiced SKY or a group that practiced armchair relaxation [37]. It was observed that there were no safety issues. Compliance was good. The data suggest that participants in the SKY group, but not the control group, lowered their degree of anxiety, depression, and stress and also increased their degree of optimism. The participants in the SKY group experienced the practices as a positive event that induced beneficial effects. This has heuristic value. The data obtained suggests that wellness can be improved by the practice of SKY.

In a study from the United States, with the SKY school programme (which combines breath-based techniques and a social-emotional learning curriculum), it was seen that students reported fewer poor mental health days at follow-up. Lower hair cortisol (a marker of chronic stress) and higher hair oxytocin levels (a measure of social affiliation) were seen within a period of nine months. This study emphasizes that SKY improves social affiliation and hypothalamic-pituitary-adrenal-axis (HPA) axis regulation [57].

This breath work-based life skills workshop, in which SKY is taught, and the subsequent practice of SKY have shown to be beneficial in teenagers [57-59], adults [60,61], post-menopausal women [49], and medical professionals [25,54] in various studies. The benefits of online use of SKY have also been proven and found feasible during the COVID-19 and post-COVID era [25,29,32,62]. Thus, there are reports of benefits of SKY in multiple aspects of health from across the globe.

Discussion

This review reveals consistent evidence supporting the beneficial effect of SKY among various populations, systems of the human body in health and disease. EEG studies have shown that SKY shifts the frequency of electrical brain activity to a higher frequency range [2], enhances interhemispheric synchronization [2], and increases pre-attentive processing [9]. This translates to better task performance [5], better workload tolerance/increased capacity for multitasking [5], improved working memory [6,10], better cognitive ability [10], and better attention/concentration [10].

It is found to decrease stress [4,7,14,19,30,31,39,54,63,64], depression [18,33-34,36,63-64], anxiety [13-14,18,33-34,63-64], and improve resilience [57] among adolescents and adults. It has also shown to decrease craving [28] in addiction and substance abuse. Though the sample size of this study was small, these findings have public health implications and need to be widely studied.

Among healthy adults, it has shown to benefit cardiac autonomic tone [11-13], cardiorespiratory coupling [14,15], and can be a useful non-pharmacological intervention to decrease cardiovascular risk. In the context of the recent increase in sudden cardiac death at a younger age, this holds importance. Across studies, SKY was shown to benefit in post-traumatic stress disorder [24,29-32], substance abuse [26,28], and addictions [41,51] and improve social connectedness [59].

In challenging professions like military veterans and health care professionals during the pandemic, SKY has been shown to improve well-being and prevent burnout [25]. In persons living with chronic diseases like diabetes and HIV, it has shown to improve QoL [33,47-50]. These improvements were consistently seen across diverse racial and demographic groups. These findings underscore the use of SKY as an adjunct in the prevention and treatment of lifestyle diseases and others. More RCTs with longer follow-ups need to be undertaken to see the effects and establish how it works. The feasibility studies done [24,29,32,39] make it evident that such studies are possible on a larger scale. It can serve as a complementary strategy alongside pharmacological treatment in chronic diseases, as it not only improves key parameters but also has a beneficial effect on QoL, mood changes, perceived stress, and emotion regulation. It is important to note that none of the studies have reported any adverse effects of SKY, making it safe to use. Table 1 summarizes the effects of SKY on various organ systems and conditions.

**Table 1:** Effects of SKY on various organ systems and conditions SKY: Sudarshan Kriya Yoga; PTSD: Post-traumatic stress disorder; HPA: Hypothalamic-pituitary-adrenal-axis.

Organ system/condition	Findings	Relevant references
Central nervous system	Interhemispheric synchronization, increase in gamma and beta waves, change in amplitude of brain waves related to state of relaxation, improved concentration, restful alertness, improved information processing, improved performance, reduced error, better work load tolerance and multitasking, improved working memory, enhanced pre-attentive processing, and improved cognitive abilities	[2-10]
Cardiovascular and respiratory system	Positive effect on cardiac autonomic functions, heart rate variability, cardiac autonomic tone, sympathovagal balance, psychophysiologic relaxation, and enhanced cardiovascular and respiratory synchronization	[11-19]
Oxidative stress markers	Better antioxidant state, lower levels of blood lactate, malondialdehyde adducts, urea, increased levels of superoxide dismutase, catalase, glutathione, glutathione peroxidase, and glutathione reductase	[20-23]
Depression/anxiety/PTSD/mental health	Improves depression, perceived stress, emotion regulation, life satisfaction, social connectedness, self-esteem, conscientiousness, and gratitude; reduces anxiety, sadness, and fatigue	[13,14,24-37]
Addictions and substance abuse	Decreases substance use cravings; improves physical and social functioning, mental and emotional well-being, and control of tobacco habit	[26,28,40]
Sleep and insomnia	Improves insomnia, nocturnal sleep, and daytime wakefulness	[25,41,42]
Genetic modulation	Reduces mRNA expressions of pro-inflammatory genes, increases expression of antioxidative genes, and increases telomere length	[43-46]
Quality of life	Improves quality of life	[29,33,37,47,48,50,51]
Immune function	Increases natural killer cell count, increases expression of anti-inflammatory genes (SOD and glutathione peroxidase), and decreases expression of pro-inflammatory genes (IL 1β, IL6, and TNF)	[23,40,44]
Diabetes mellitus	Improved glycemic control, quality of life, cardiac autonomic functions, improved dietary practices, and adherence to medication	[16,17,33,47,52,53]
Wellness and optimism	Reduces stress, poor mental health days; improves optimism, social affiliation, and HPA axis regulation	[25,30,31,37,54-57]

Research is also needed to establish the diseases for which SKY can serve as an adjunct to pharmacological treatment as it has been shown to affect gene expression [43-46]. Seeing its wide availability over and now online in the post-COVID era, practical implementation in real-world settings can empower persons with increased awareness to adopt suitable health-benefiting behaviors and provide a support group to continue. Persons with diabetes practicing SKY were more successful in improving dietary practices and medication adherence compared to those not practicing [53]. Though this study was small, it opens up ground for future research.

Nonetheless, the findings suggest that breathing practices like SKY could have systemic effects, although greater methodological rigor is needed to confirm their multi-organ impact. SKY increases parasympathetic activity [11-18] and thus calms the stress response. EEG studies show it changes the amplitude of brain signals to create restful alertness [3]. It enhances well-being [13,14,28,37,56,58], mood [18,33-34,36,63,64], attention [5-6], mental focus [7], multitasking, and stress tolerance [4,7,14,19,30-31,39,54,63,64]. Other mechanisms include metabolic [14-15,20-23,27,53,57], epigenetic [43-46], anti-inflammatory [44], and immunological [23]. Evidence has built up over the last three decades to consider SKY as a safe, inexpensive, feasible, and effective adjunct in the management of anxiety, depression [18], PTSD [19], stress-related medical illnesses, addictions, substance abuse, and rehabilitation [64]. Sky improves QoL in chronic diseases like diabetes [33,47], HIV [48], opioid dependence [50], alcohol dependence [51], and healthy persons [11-15,37]. The effects of SKY on gene transcription [43-46] could be another important mechanism by which the practice of SKY prevents the manifestation of disease symptoms.

Body, mind, and spirit are interconnected [65,66]. The development of many diseases is an interplay between genetic predisposition (non-modifiable) and environmental factors (modifiable). Though genes cannot be modified, their expression can be altered to promote health. The practice of SKY has been shown to affect gene expression [43-46].

The Sanskrit word for health is "Swasthya." "Swa" means self, and "Sthya" means established; being healthy (Swasthya) means we are anchored and connected to our true self. Human existence has various levels - body, breath, mind, intellect, memory, ego, and self [67]. These levels of our existence are interconnected. By practicing SKY, these layers of our existence are synchronized, and we become aware of these layers. This could help in preventing the progression of the disease. In an RCT, it was seen that in patients with diabetes practicing SKY, the progression of cardiac autonomic neuropathy was less compared to the group not practicing SKY [16]. Also, by bestowing restful alertness [3], SKY makes practitioners more aware of themselves and their surroundings. Practice of SKY has shown to reduce drug abuse craving [28] and confer better dietary choices and adherence to medicines [53]. The stress-relieving principles and life skills discussed in this workshop with the restful alertness and optimism the practice of SKY endows could help with anxiety, depression, stress, QoL, social skills, and making right lifestyle choices in chronic diseases.

These studies have their challenges. Though it is inexpensive and easy to learn, the willingness of the person to practice SKY daily and attend weekly follow-up for group long SKY practice requires patient willingness and involvement. Also, funding for such research lacks the monetary push from pharmaceutical companies. It is important that physicians are aware of the research evidence of the benefits of SKY so that more research can be done to generate evidence and the benefits can be applied to patients.

## Conclusions

Disease development involves a complex interplay between non-modifiable (genetics, ethnicity, and age) and modifiable (dietary habits, physical activity, thought management, behavior, and response) risk factors. SKY and related practices modulate gene expression and positively affect physiological processes (shown in HRV and EEG studies), stress reduction, mental health, immunity, autonomic function, sleep, QoL, self-awareness, thought, and response, which translate into better health over time. SKY has been shown to be a safe, effective, low-cost, and scalable intervention in a variety of conditions, as treatment to prevent progression or as an adjunct to pharmacological treatment.

However, more long-term and larger RCTs are required to establish it as an adjunct therapy in chronic diseases and in the prevention of unhealthy behaviors leading to chronic diseases. With this background, it is imperative to do long-term research on this promising breath-mind-body technique.
